# Shared Genetic Architecture Between Schizophrenia and Anorexia Nervosa: A Cross-trait Genome-Wide Analysis

**DOI:** 10.1093/schbul/sbae087

**Published:** 2024-06-07

**Authors:** Zheng-An Lu, Alexander Ploner, Andreas Birgegård, Eating Disorders Working Group of the Psychiatric Genomics Consortium, Roger Adan, Lars Alfredsson, Tetsuya Ando, Ole Andreassen, Jessica Baker, Andrew Bergen, Wade Berrettini, Andreas Birgegård, Joseph Boden, Ilka Boehm, Vesna Boraska Perica, Harry Brandt, Gerome Breen, Julien Bryois, Katharina Buehren, Cynthia Bulik, Roland Burghardt, Matteo Cassina, Sven Cichon, Jonathan Coleman, Roger Cone, Philippe Courtet, Steven Crawford, Scott Crow, James Crowley, Unna Danner, Oliver Davis, Martina de Zwaan, George Dedoussis, Janiece DeSocio, Danielle Dick, Dimitris Dikeos, Christian Dina, Monika Dmitrzak-Weglarz, Elisa Docampo, Laramie Duncan, Karin Egberts, Stefan Ehrlich, Geòrgia Escaramís, Tõnu Esko, Xavier Estivill, Anne Farmer, Angela Favaro, Fernando Fernández-Aranda, Krista Fischer, Manuel Föcker, Lenka Foretova, Andreas Forstner, Monica Forzan, Christopher Franklin, Steven Gallinger, Ina Giegling, Paola Giusti-Rodríguez, Fragiskos Gonidakis, Scott Gordon, Philip Gorwood, Monica Gratacos Mayora, Jakob Grove, Sébastien Guillaume, Yiran Guo, Hakon Hakonarson, Katherine Halmi, Ken Hanscombe, Konstantinos Hatzikotoulas, Joanna Hauser, Johannes Hebebrand, Sietske Helder, Stefan Herms, Beate Herpertz-Dahlmann, Wolfgang Herzog, Anke Hinney, L John Horwood, Christopher Hübel, Laura Huckins, James Hudson, Hartmut Imgart, Hidetoshi Inoko, Vladimir Janout, Susana Jiménez-Murcia, Craig Johnson, Jennifer Jordan, Antonio Julià, Gursharan Kalsi, Deborah Kaminská, Allan Kaplan, Jaakko Kaprio, Leila Karhunen, Andreas Karwautz, Martien Kas, Walter Kaye, James Kennedy, Martin Kennedy, Anna Keski-Rahkonen, Kirsty Kiezebrink, Youl-Ri Kim, Lars Klareskog, Kelly Klump, Mikael Landén, Janne Larsen, Stephanie Le Hellard, Virpi Leppä, Dong Li, Paul Lichtenstein, Lisa Lilenfeld, Bochao Danae Lin, Jolanta Lissowska, Jurjen Luykx, Mario Maj, Sara Marsal, Nicholas Martin, Manuel Mattheisen, Morten Mattingsdal, Sarah Medland, Andres Metspalu, Ingrid Meulenbelt, Nadia Micali, Karen Mitchell, James Mitchell, Alessio Maria Monteleone, Palmiero Monteleone, Preben Bo Mortensen, Melissa Munn-Chernoff, Benedetta Nacmias, Marie Navratilova, Ioanna Ntalla, Catherine Olsen, Roel Ophoff, Leonid Padyukov, Jacques Pantel, Hana Papezova, Richard Parker, John Pearson, Nancy Pedersen, Liselotte Petersen, Dalila Pinto, Kirstin Purves, Anu Raevuori, Nicolas Ramoz, Ted Reichborn-Kjennerud, Valdo Ricca, Samuli Ripatti, Stephan Ripke, Franziska Ritschel, Marion Roberts, Dan Rujescu, Filip Rybakowski, Paolo Santonastaso, André Scherag, Stephen Scherer, Ulrike Schmidt, Nicholas Schork, Alexandra Schosser, Jochen Seitz, Lenka Slachtova, P Eline Slagboom, Margarita Slof-Op 't Landt, Agnieszka Slopien, Sandro Sorbi, Michael Strober, Patrick Sullivan, Beata Świątkowska, Jin Szatkiewicz, Elena Tenconi, Laura Thornton, Alfonso Tortorella, Janet Treasure, Artemis Tsitsika, Marta Tyszkiewicz-Nwafor, Annemarie van Elburg, Eric van Furth, Tracey Wade, Gudrun Wagner, Hunna Watson, Thomas Werge, David Whiteman, Elisabeth Widen, D Blake Woodside, Shuyang Yao, Zeynep Yilmaz, Eleftheria Zeggini, Stephanie Zerwas, Stephan Zipfel, Gerome Breen, Cynthia Bulik, Cynthia M Bulik, Sarah E Bergen

**Affiliations:** Department of Medical Epidemiology and Biostatistics, Karolinska Institutet, Stockholm, Sweden; Department of Medical Epidemiology and Biostatistics, Karolinska Institutet, Stockholm, Sweden; Department of Medical Epidemiology and Biostatistics, Karolinska Institutet, Stockholm, Sweden; Department of Medical Epidemiology and Biostatistics, Karolinska Institutet, Stockholm, Sweden; Department of Psychiatry, University of North Carolina at Chapel Hill, Chapel Hill, NC, USA; Department of Nutrition, University of North Carolina at Chapel Hill, Chapel Hill, NC, USA; Department of Medical Epidemiology and Biostatistics, Karolinska Institutet, Stockholm, Sweden

**Keywords:** genetic, architecture, schizophrenia, anorexia, nervosa, Mendelian randomization, pleiotropy, GWAS, polygenic overlap

## Abstract

**Background and Hypothesis:**

Schizophrenia (SCZ) and anorexia nervosa (AN) are 2 severe and highly heterogeneous disorders showing substantial familial co-aggregation. Genetic factors play a significant role in both disorders, but the shared genetic etiology between them is yet to be investigated.

**Study Design:**

Using summary statistics from recent large genome-wide association studies on SCZ (*N*_cases_ = 53 386) and AN (*N*_cases_ = 16 992), a 2-sample Mendelian randomization analysis was conducted to explore the causal relationship between SCZ and AN. MiXeR was employed to quantify their polygenic overlap. A conditional/conjunctional false discovery rate (condFDR/conjFDR) framework was adopted to identify loci jointly associated with both disorders. Functional annotation and enrichment analyses were performed on the shared loci.

**Study Results:**

We observed a cross-trait genetic enrichment, a suggestive bidirectional causal relationship, and a considerable polygenic overlap (Dice coefficient = 62.2%) between SCZ and AN. The proportion of variants with concordant effect directions among all shared variants was 69.9%. Leveraging overlapping genetic associations, we identified 6 novel loci for AN and 33 novel loci for SCZ at condFDR <0.01. At conjFDR <0.05, we identified 10 loci jointly associated with both disorders, implicating multiple genes highly expressed in the cerebellum and pituitary and involved in synapse organization. Particularly, high expression of the shared genes was observed in the hippocampus in adolescence and orbitofrontal cortex during infancy.

**Conclusions:**

This study provides novel insights into the relationship between SCZ and AN by revealing a shared genetic component and offers a window into their complex etiology.

## Introduction

Schizophrenia (SCZ) is a severe and clinically heterogeneous psychiatric disorder characterized by hallucinations, delusions, negative symptoms, disorganized behavior, and cognitive impairment.^1^ Anorexia nervosa (AN) is a serious psychiatric disorder characterized by very low body weight, restriction of energy intake, extreme concern over body shape and weight, and elevated mortality.^2–4^ Both SCZ and AN often follow a chronic course and have poor outcomes due to extensive comorbidities and lack of effective treatments, posing substantial challenges for patients and their families.^5^

Despite distinct clinical features, a relationship between SCZ and AN has frequently been observed. First, symptoms of SCZ and AN sometimes co-occur: distorted perceptions of body shape in AN patients sometimes have delusional features as in psychosis,^6^ and delusions about food and body image in some SCZ patients can lead to food restriction typical of AN.^6^ Moreover, AN can arise in the prodromal period of SCZ, and psychotic experiences during childhood are associated with a higher future risk of disordered eating behaviors.^6,7^ Symptoms of AN are up to 13 times more prevalent in SCZ patients, and individuals with AN show 6 times greater risk of also having SCZ.^6,8^

Genetic factors play an important role in both disorders, with the heritability estimated at 60%–80% for SCZ and 50%–60% for AN.^9,10^ Recent genome-wide association studies (GWASs) identified 287 loci for SCZ and 8 loci for AN,^11,12^ and some preliminary studies have explored the genetic association between SCZ and AN. One population-based study in Sweden and Denmark revealed a substantial familial co-aggregation of SCZ and AN, indicating shared genetic risk.^8^ Furthermore, genetic liability to SCZ was associated with distinct phenotypes within AN, and vice versa: AN patients with a family history of SCZ demonstrated higher somatic and mental health burden but lower AN symptom severity, and SCZ patients with higher genetic liability to AN exhibited lower risk for cardiovascular and metabolic disorders.^13^ At the genomic variant level, in a cross-disorder GWAS meta-analysis, 6 of 23 loci with the broadest pleiotropic effects were confidently associated with both SCZ and AN, consistent with the high single nucleotide polymorphism (SNP)-based genetic correlation (*r*_g_ = .19–.29).^3,12,14^ Altogether, these findings indicate genetic overlap between SCZ and AN and support deeper investigation of their shared genetic etiology.

SCZ and AN are both highly polygenic disorders, with numerous genetic variants conferring small effects contributing to overall liability.^11,12^ Despite growing sample sizes for GWASs, a large proportion of genomic variants influencing SCZ and AN has not yet been identified, and their causal relationship, the extent of their polygenic overlap, and the specific shared loci remain largely unknown. Furthermore, the effect directions of the shared loci between SCZ and AN can be either concordant or discordant for the 2 disorders, which cannot be captured by traditional genetic correlation methods.^15^ Some recently developed tools can solve the problem and reveal more detailed shared genetic architecture: MiXeR can estimate the number of shared variants irrespective of the direction of effects, and the conditional/conjunctional false discovery rate (cond/conjFDR) approach can identify shared loci with a mixture of effect directions.^16^

The current study has 4 aims designed to deepen our understanding of the shared etiology and biology between SCZ and AN: (1) to explore the causal relationship between SCZ and AN using genetically informative approaches; (2) to quantify polygenic overlap between SCZ and AN using MiXeR; (3) to identify disorder-specific novel and shared genomic loci for SCZ and AN using cond/conjFDR; (4) to reveal biological mechanisms shared between SCZ and AN.

## Materials and Methods

### Participant Samples

GWAS summary statistics (*P* values and beta scores) for SCZ and AN were acquired from the Psychiatric Genomics Consortium (PGC): the SCZ dataset was based on 53 386 cases with SCZ or schizoaffective disorder and 77 258 controls from 75 studies^11^; the AN dataset was based on 16 992 AN cases and 55 525 controls from 33 studies.^12^ Most participants in the constituent studies were of European ancestry.

### Statistical Analyses

#### Conditional Q*-*Q Plots

To visualize the cross-trait genetic enrichment between SCZ and AN, we constructed conditional quantile-quantile (Q-Q) plots. Conditional Q-Q plots contrast genetic associations of a primary disorder across all SNPs with SNP subgroups defined by increasingly stringent *P* value thresholds for association with a secondary disorder (*P* value < .1, *P* value <.01, *P* value <.001). Successive upward deflection of the conditional Q-Q curves of the primary disorder with increasing strength of association with the secondary disorder indicates polygenic overlap at the common variant level.

#### Two-Sample Mendelian Randomization Analysis

To evaluate the causal relationship between SCZ and AN, we performed 2-sample bidirectional Mendelian randomization (MR) analyses based on GWAS summary statistics of SCZ and AN, by leveraging 5 different MR methods.^11,12^ All statistical analyses were performed using the R package TwoSampleMR version 0.5.6 and MRPRESSO version 1.0. For details, see supplementary methods.

#### MiXeR Analysis

To quantify polygenic overlap between SCZ and AN, we applied a causal mixture model to GWAS summary statistics, using MiXeR v1.3 (https://github.com/precimed/mixer). MiXeR can estimate the number of trait-influencing variants with nonzero effects, as well as the number of shared variants between disorders, irrespective of the direction of effect.^15^ MiXeR allows for the discovery of extensive polygenic overlap when 2 disorders show insignificant genetic correlation with a mixture of variants with concordant and discordant effect directions.^17^ Genetic effects for 2 disorders are divided into 4 components: (1) SNPs associated with both disorders, (2) SNPs associated with neither disorder, and (3 + 4) 1 component each for SNPs specific to each disorder; the estimated number of variants in each component is visualized as a Venn diagram.^15^ Genetic association and polygenic overlap are summarized as genetic correlation and Dice coefficient (DC, estimated proportion of shared variants out of all variants with nonzero effects), respectively.^15^ We also conducted MiXeR analysis for AN and other somatic (ie, height) and psychiatric traits (ie, obsessive-compulsive disorder and anxiety disorder) to offer comparisons for our main analysis.^18–20^ For details, see supplementary methods.

#### Conditional/Conjunctional FDR Analysis

We applied condFDR for genomic locus detection. CondFDR is an extension of the conventional FDR in an empirical Bayesian framework, allowing inference on genetic variants associated with a primary disorder conditional on their genetic association with a secondary disorder.^16^ It is defined as the probability that an SNP has a null association with the primary disorder conditional on the fact that the *P* value for the secondary disorder is as small or smaller than the observed *P* value. We used conjFDR, an extension of condFDR to identify shared genomic loci between SCZ and AN. ConjFDR is defined as the maximum of the 2 condFDR values produced by exchanging the roles of primary and secondary disorder in condFDR analysis, and provides a conservative FDR estimate for a genetic variant to be jointly associated with both disorders. Cond/conjFDR framework leverages the statistical power from 2 GWAS studies to boost the discovery of genomic loci.^16^ Significance thresholds were set at condFDR <0.01 and conjFDR <0.05, as recommended.^21^ Given the limited power of AN GWAS, we also used a more relaxed significance level of conjFDR <0.1 to identify shared loci, which was employed to boost variant discovery in previous research.^22^ All cond/conjFDR statistical analyses were performed using the R package cfdr.pleio version 0.0.0.9100 available online (https://github.com/alexploner/cfdr.pleio). For details, see supplementary methods.

#### Definition of Genomic Loci, Functional Annotation, Gene Mapping, and Expression Analysis

We defined genomic loci following the Functional Mapping and Annotation (FUMA) protocol recommendations.^23^ Independent significant SNPs were defined as SNPs independent with each other at *r*^2^ < 0.6 and with condFDR <0.01 or conjFDR <0.05/conjFDR <0.1. A subgroup of independent significant SNPs in approximate linkage equilibrium at *r*^2^ < 0.1 were selected as lead SNPs. Physically overlapping genomic loci were merged as 1 locus (<250 kb apart), and the SNP with the lowest cond/conjFDR was selected as the lead SNP for the locus. Candidate SNPs in each locus were defined as those in linkage disequilibrium at *r*^2^ > 0.6 with any of the independent significant SNPs, and having a condFDR <0.01 or conjFDR <0.05/conjFDR <0.1.

FUMA v1.5.0 (https://fuma.ctglab.nl/) was utilized to perform functional annotation and gene mapping. ANNOVAR, combined annotation-dependent depletion (CADD) scores, RegulomeDB scores, and chromatin states were employed to functionally annotate all candidate SNPs in linkage disequilibrium at *r*^2^ > 0.6 with one of the independently significant SNPs in conjFDR analyses. To identify shared genes, we employed positional mapping, expression quantitative trait locus (eQTL) mapping, and chromatin interaction mapping to map genes to candidate SNPs in shared loci. By querying the GTEx v8 database, we performed Multi-marker Analysis of GenoMic Annotation (MAGMA) tissue expression analysis to determine the pattern of expression across tissues.^23,24^

To explore whether shared genes were significantly enriched in specific biological pathways, we applied gene ontology (GO) analysis to all genes mapped to the shared loci.^25^ GO analysis was performed with the R package clusterProfiler version 3.18.1, and the results were visualized using the R package enrichplot version 1.10.2.

To visualize the spatiotemporal genetic expression pattern, we generated heatmaps for the expression differences between shared genes vs all background genes across 16 brain regions and 11 developmental time points based on BrainSpan RNA sequencing data.^26^ BrainSpan captures the genetic expression levels in different brain regions from 8 post-conceptual weeks to 40 years in healthy humans.^26^ For a detailed description, see supplementary methods.

## Results

### Cross-trait Polygenic Overlap Between SCZ and AN

**Fig. 1.**
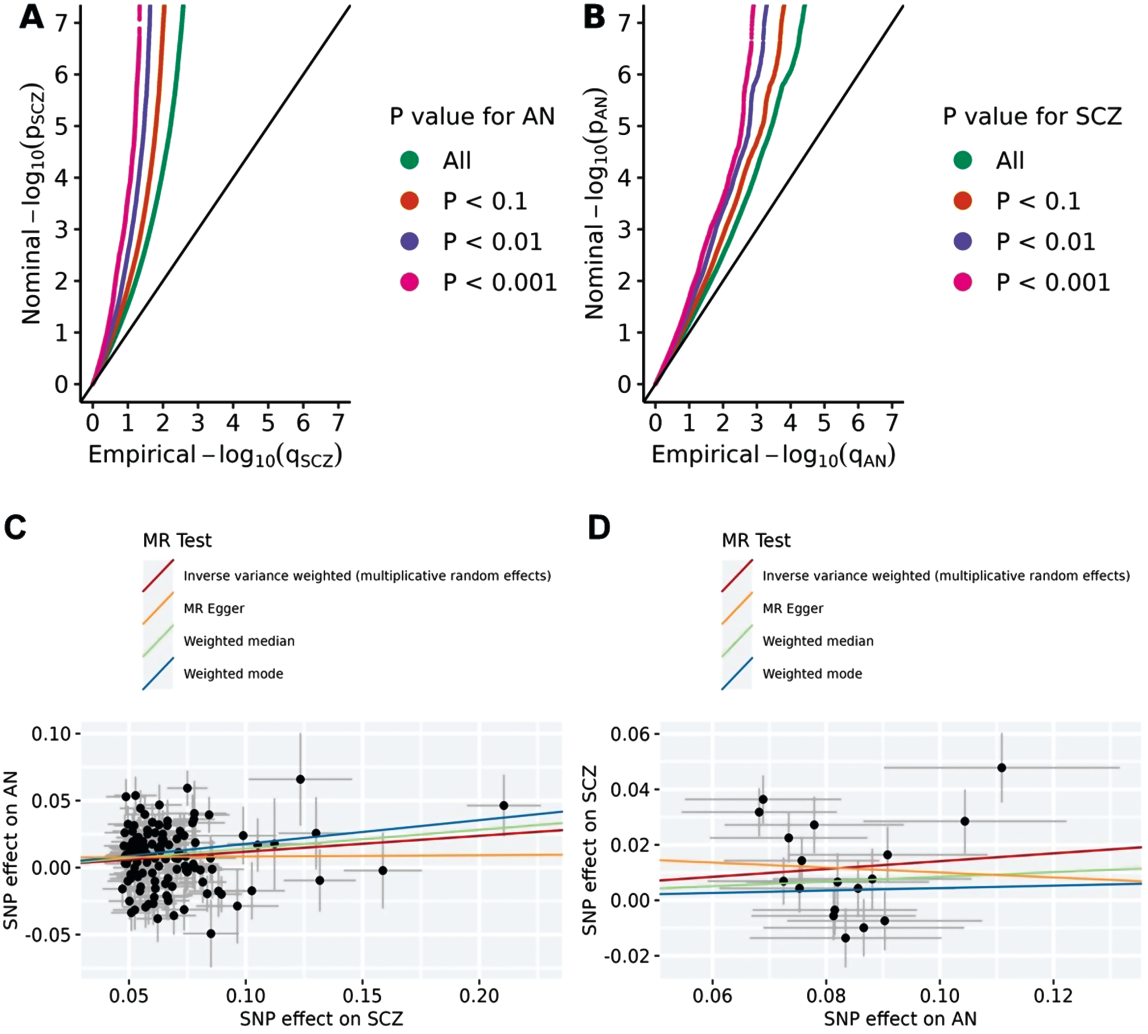
Conditional quantile-quantile (Q-Q) plots and Mendelian randomization scatter plots for the associations between schizophrenia (SCZ) and anorexia nervosa (AN). The conditional quantile-quantile plot of nominal vs empirical −log_10_ p values for SCZ as a function of significant association with AN at *P* value <.1, *P* value <.01, and *P* value <.001 (A), and vice versa (B). The black diagonal line indicates the global null hypothesis. Scatter plots for effects of SCZ and AN for instrumental variants of SCZ (panel C, *n* = 127), and AN (panel D, *n* = 18). Lines represent the regression of SNP effects for exposure (SCZ for A and AN for B) on the SNP effects for outcome (AN for A and SCZ for B), corresponding to a meta-analysis using random-effects inverse variance weighted, MR-Egger regression, weighted-mode, and weighted-median methods. *Note*: MR, Mendelian randomization.

We observed enrichment in genetic association with SCZ as a function of the significance of association with AN, as indicated by the successive leftward deflection in the SCZ Q-Q plot as the *P* value for AN decreases (figure 1). The same was observed in the reverse direction: SNPs passing more stringent *P* value thresholds for SCZ generally demonstrated stronger associations with AN (figure 1).

For MR analyses, a total of 127 and 18 SNPs were selected as instrumental variables for SCZ and AN, respectively (supplementary table S1). We found some evidence for a weak positive causal effect of SCZ on AN across multiple methods (odds ratio [OR] = 1.11–1.19, supplementary table S1), as well as for a comparable effect of AN on SCZ (OR = 1.05–1.15, supplementary table S2), with the exception of MR-Egger, which provided a flat (OR = 1.01) risk estimate for SCZ on AN and a reversed effect for AN on SCZ (OR = 0.91, supplementary table S2). These results reveal a suggestive but inconclusive bidirectional causal relationship between SCZ and AN. For details, see supplementary results.

**Fig. 2.**
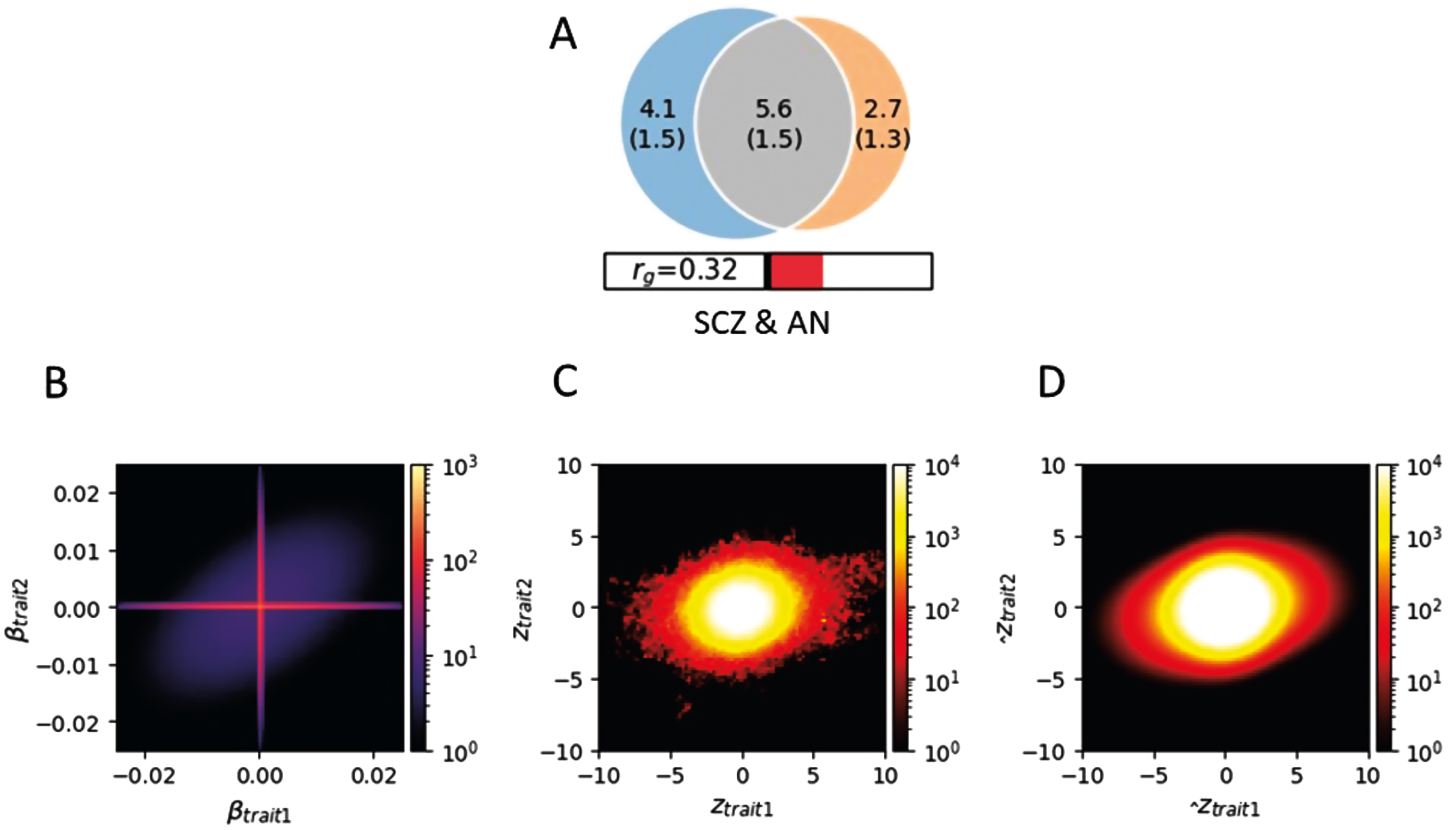
Polygenic overlap between schizophrenia (SCZ) and anorexia nervosa (AN). (A) The Venn diagram illustrates the estimated number of non-null variants shared between SCZ and AN, and specific to each disorder: numbers in the circle indicate the quantity (standard error) of genetic variants in thousands. The circle on the left represents SCZ, the circle on the right represents AN, and the center part represents overlap. The size of the circle indicates polygenicity, with a larger circle reflecting greater polygenicity. The estimated genetic correlation is also shown below the Venn diagram, with an accompanying directional scale (shading to the right indicates positive correlation). (B) The density plot for additive causal effects that underlie model prediction. (C) The density plot for observed GWAS signed test statistics. (D) The corresponding density plot predicted from the fitted MiXeR model. Trait1 = SCZ; Trait2 = AN. *Note*: GWAS, genome-wide association study.

Consistent with the Q-Q plots, MiXeR identified considerable polygenic overlap between SCZ and AN (DC = 62.2%, *r*_g_ = .32, figure 2 and supplementary table S5), which is significantly greater than the overlap between AN and comparison traits (AN vs height: DC = 14.70%, *r*_g_ = .00; AN vs OCD: DC = 17.20%, *r*_g_ = .28; AN vs ANX: DC = 33.38%, *r*_g_ = .20, supplementary figure S2 and supplementary table S6). Furthermore, of 9704 variants influencing SCZ and 8272 variants influencing AN, 5617 were shared between SCZ and AN, and 69.61% of the shared variants influenced SCZ and AN in the same direction (figure 2 and supplementary table S5). Our model had an acceptable fit, as demonstrated by the minimal deviation of model-predicted conditional Q-Q plots from the observed ones (supplementary figure S3).

### Novel Disorder-Specific Genomic and Shared Loci Between SCZ and AN

**Fig. 3.**
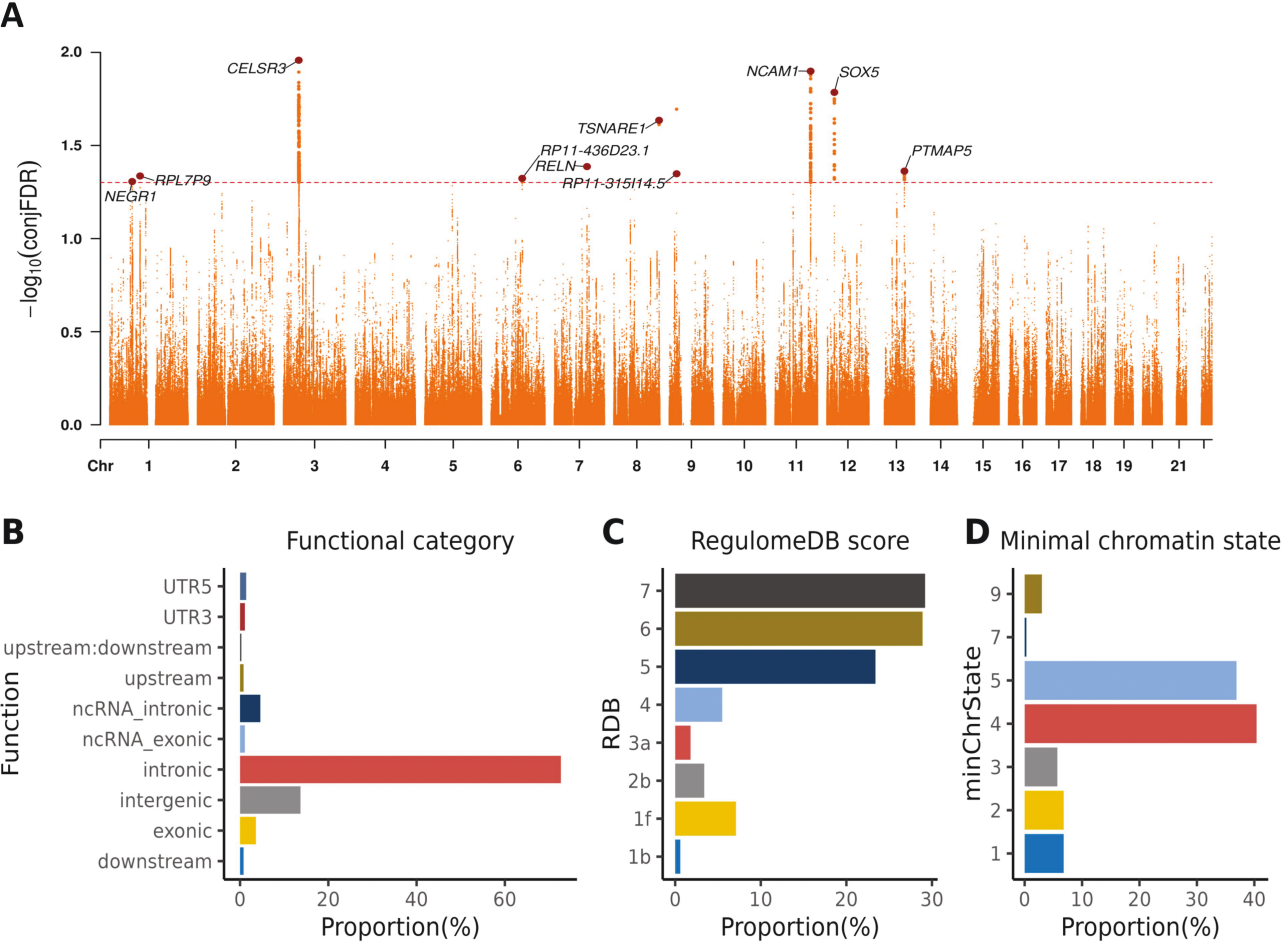
Common genomic loci jointly associated with schizophrenia and anorexia nervosa at conjunctional false discovery rate (conjFDR) <0.05. (A) A Manhattan plot with −log_10_-transformed conjFDR values for each SNP on the *y*-axis and chromosomal positions along the *x*-axis. The dashed horizontal line represents the threshold for significant shared associations. Independent lead SNPs are marked by large dots. The gene nearest to each shared locus is annotated. (B) The distribution of functional consequences with SNPs annotated using ANNOVAR. (C) The distribution of likely regulatory functions annotated using RegulomeDB. (D) The distribution of the minimum chromatin states, annotated based on 15 categorical states, with lower states indicating higher accessibility to regulatory elements.

At condFDR <0.01, we identified 255 loci associated with SCZ conditional on AN; of these, 222 were identified in original SCZ GWAS or subsequent GWAS analyses, producing a total of 33 novel SCZ loci (supplementary table S7). In contrast, we identified 12 loci for AN at condFDR <0.01, of which 6 were identified in the original AN GWAS, yielding a total of 6 novel loci for AN (supplementary table S8). Additionally, conjFDR analysis identified 10 and 42 genomic loci jointly associated with SCZ and AN at conjFDR <0.05 and conjFDR <0.1, respectively (table 1, figure 3, and supplementary table S9). All shared loci showed concordant effect directions for SCZ and AN at conjFDR <0.05, but 3 shared loci showed discordant effect directions (locus 10, 13, 15) at conjFDR <0.1 (table 1 and supplementary table S9).

**Table 1.** Genomic Loci Jointly Associated With Schizophrenia and Anorexia Nervosa at conjFDR <0.05^a^,*,

Locus	Chr	Lead SNP	A1	A2	Nearest Gene	Functional Category	CADD	RDB	min ChrState	BETA for SCZ	*P* Value for SCZ	BETA for AN	*P* Value for AN	ConjFDR
1	1	rs12049457	A	G	NEGR1	Intronic	1.756	5	2	0.042	1.78 × 10^−6^	0.058	3.29 × 10^−5^	0.049
2	1	rs77663034	C	T	RPL7P9	Intergenic	4.164	5	7	−0.061	1.63 × 10^−4^	−0.113	9.78 × 10^−6^	0.046
3	3	rs9878063	T	G	CELSR3	Intronic	13.260	4	4	−0.062	5.00 × 10^−6^	−0.135	8.77 × 10^−11^	0.011
4	6	rs2388334	A	G	RP11-436D23.1	ncRNA_intronic	5.756	7	5	−0.032	1.80 × 10^−4^	−0.056	2.82 × 10^−5^	0.048
5	7	rs10254364	G	A	RELN	Intergenic	2.652	6	5	−0.033	1.12 × 10^−4^	−0.065	1.98 × 10^−6^	0.041
6	8	rs4129585	A	C	TSNARE1	Intronic	6.646	/	5	0.075	5.11 × 10^−18^	0.0593	9.42 × 10^−6^	0.023
7	9	rs11795350	A	G	RP11-315I14.5	Intergenic	2.079	7	4	0.092	1.75 × 10^−6^	0.131	2.52 × 10^−5^	0.045
8	11	rs4144893	G	A	NCAM1	Intronic	1.115	7	5	−0.051	3.46 × 10^−6^	−0.078	4.06 × 10^−6^	0.013
9	12	rs4554988	G	A	SOX5	Intergenic	8.800	7	5	−0.038	1.09 × 10^−5^	−0.067	1.36 × 10^−6^	0.016
10	13	rs9546027	T	A	PTMAP5	Intergenic	2.057	7	9	0.035	1.05 × 10^−4^	0.062	2.31 × 10^−5^	0.043

^a^A1, allele 1; A2, allele 2; CADD, combined annotation-dependent depletion score; Chr, chromosome; ConjFDR, conjunctional false discovery rate; minChrState, minimum chromatin state; ncRNA, noncoding RNA; RDB, RegulomeDB score; UTR, untranslated region.

*The effect sizes are provided with A2 as the reference allele.

### Functional Annotation, Gene Mapping, and Enrichment Analysis

Functional annotation of the 366 candidate SNPs in the 10 shared loci at conjFDR <0.05 revealed that most of the SNPs were located in intergenic (13.7%) or intronic (72.7%) regions, with only 13 SNPs (3.6%) in exonic regions (figure 3 and supplementary table S9). Among all candidate SNPs, 24 (6.6%) were identified as potentially deleterious with CADD score >12.37 (supplementary table S10). Moreover, a total of 25 variants had a RegulomeDB score below 2a, indicating a higher probability of regulatory function (supplementary table S10). Furthermore, the majority of candidate SNPs (355, 97.0%) had chromatin state scores between 1 and 7, suggesting open chromatin states vulnerable to regulatory elements (figure 3 and supplementary table S10). Among the 24 deleterious variants at conjFDR <0.05, 3 (rs9868809, rs12107252, and rs12629759) had a RegulomeDB score of 1f (supplementary table S9), suggesting their regulatory effects via eQTL + transcription factor (TF)-binding/DNase peak mechanisms. After querying the GTEx v8 database (https://gtexportal.org/), we found the variants were eQTLs for *NCKIPSD* and *WDR6* in multiple brain regions. Functional annotations for candidate SNPs in the shared loci at conjFDR <0.1 are presented in supplementary table S11.

**Fig. 4.**
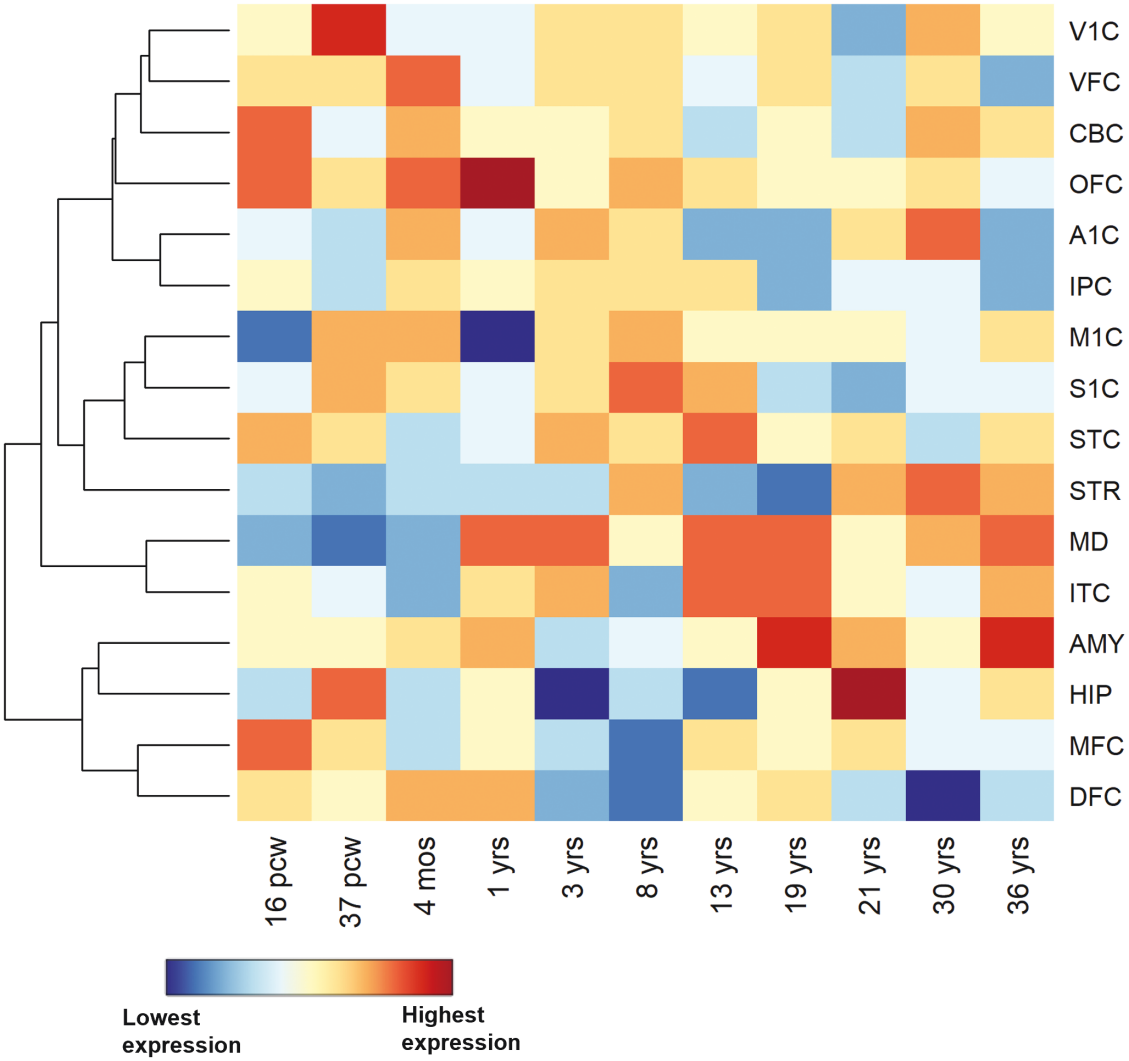
Spatiotemporal expression difference between shared genes between schizophrenia and anorexia nervosa and background genes across 16 brain regions and 11 developmental time points. The mean expression difference between 127 genes mapped to shared loci and 52 376 background genes with nonzero expression in the BrainSpan dataset is illustrated in color ranging from red (high expression) to blue (low expression). The gene expression values are log_2_-transformed, and the differences in transformed expression values are centered and scaled across 16 brain regions at each time point. The color represents the relative differential genetic expression in a brain region among all 16 brain regions at a specific time point. Brain regions are clustered using unsupervised hierarchical cluster analysis. *Note*: A1C, primary auditory cortex; ACC, anterior cingulate cortex; AMY, amygdala; CBC, cerebellum; DFC, dorsolateral prefrontal cortex; HIP, hippocampus; IPC, inferior parietal cortex; ITC, inferolateral temporal cortex; M1C, primary motor cortex; MD, mediodorsal nucleus of thalamus; MFC, medial prefrontal cortex; mos, months; OFC, orbitofrontal cortex; pcw, postconceptional weeks; S1C, primary somatosensory cortex; STC, superior temporal cortex; STR, striatum; V1C, primary visual cortex; VFC, ventrolateral prefrontal cortex; yrs, years.

FUMA v1.5.0 (https://fuma.ctglab.nl/) linked the 366 candidate SNPs from the 10 shared loci at conjFDR <0.05 between SCZ and AN to 130 protein-coding genes (supplementary table S12). A total of 37 genes were implicated by all 3 mapping methods. GO enrichment analysis indicated that the identified genes were significantly associated with the following GO terms at Benjamini-Hochberg adjusted *P* value <.05: synapse organization, regulation of synapse organization, regulation of synapse structure or activity, hyaluronan catabolic process, cellular response to light stimulus, and others (supplementary table S13 and supplementary figure S4), which were divergent from the GO terms associated with the significant SCZ loci conditional on AN at condFDR <0.01 (supplementary table S14). MAGMA tissue expression analysis revealed that candidate genes shared between SCZ and AN at conjFDR <0.05 were significantly enriched in the tibial artery (*P* value = .008) (supplementary table S12). Of the 14 brain tissues, significant enrichment of these shared genes was only observed in the cerebellar hemisphere (*P* value = .018), cerebellum (*P* value = .028), and pituitary (*P* value = .047) (supplementary table S15). Particularly, high expression of these shared genes was observed in the hippocampus in adolescence and orbitofrontal cortex during infancy (figure 4). Genes mapped to the 42 shared loci at conjFDR <0.1 and the GO terms associated with the 3 discordant shared loci are presented in supplementary tables S16 and S17.

## Discussion

Our study is the first to probe the shared genetic profile between SCZ and AN in depth. Leveraging GWAS summary statistics, we identified a considerable polygenic overlap, tentative evidence for a bidirectional causal relationship, and 10 genomic loci jointly associated with both disorders. Our results confirm that SCZ and AN share genomic and molecular mechanisms, provide novel insights into their etiology, and offer promising targets for future experimental studies.

Conditional Q-Q plots show that genetic factors influencing SCZ are also associated with risk of AN in a dose-response manner, in agreement with the considerable polygenic overlap identified by MiXeR (62.2%) and the suggestive 2-way causal relationship seen in MR analysis. It is noteworthy that an estimated 70% of shared variants influence the 2 disorders in the same direction, which corroborates the strong positive genetic correlation identified in previous studies.^3,12,14^ These findings indicate that the partially overlapping symptoms (eg, delusional thinking and food restriction) and the high co-occurrence of SCZ and AN may be at least partially attributable to the shared genetic risk at the common variant level.^6–10^ Moreover, it is noteworthy that there is still an estimated 30% of the shared variants with discordant effects, which may to some extent explain the observed negative association between genetic liability to SCZ and the severity of AN psychopathology in some patients.^13^ In contrast, we did not identify any loci with discordant effect directions for SCZ and AN at conjFDR <0.05. This might be due to the limited GWAS power, as the proportion of discordant variants was gradually approaching the estimated value from MiXeR when we adopted incrementally more relaxed significance levels of conjFDR (supplementary figure S5).

Notably, we observed that both the polygenic overlap and *r*_g_ estimated by MiXeR for AN-SCZ were significantly greater than those for AN-OCD and AN-ANX (supplementary table S6), which is inconsistent with the fact that linkage disequilibrium (LD)-score regression-based *r*_g_ for AN-SCZ is substantially lower than *r*_g_ for AN-OCD and comparable to *r*_g_ for AN-ANX.^12^ MiXeR estimates genetic overlap irrespective of whether the SNP effect directions are consistent or incongruent for 2 disorders, thus can reveal additional shared genetic risk which traditional methods tend to ignore due to incongruent shared variants counteracting each other.^15^ As the proportion of incongruent shared variants is significantly greater for AN-SCZ than for AN-OCD and AN-ANX (supplementary table S6), the AN-SCZ correlation may be more heavily diluted when the traditional genetic correlation methods are applied. However, we cannot preclude the possibility that the low polygenic overlap between AN and OCD or ANX is due to the lower GWAS power. Our findings shed light on the potentially underestimated genetic relationship between SCZ and AN and warrant deeper investigation into the shared genetic etiology between the 2 disorders.

We detected 10 and 42 loci jointly associated with SCZ and AN at conjFDR <0.05 and conjFDR <0.1, respectively. Our study boosted the discovery by replicating 6 previously identified shared loci (locus 1, 2, 4, 6, 8, 9) and unveiling 4 novel ones (locus 3, 5, 7, 10) at conjFDR <0.05.^22^

Protein-coding genes located nearest to the identified shared loci were highly relevant to neuronal development, metabolic processes, and feeding behaviors. *NEGR1* was observed to be highly expressed in the dorsolateral prefrontal cortex of SCZ patients.^27^ Blocking *NEGR1* expression in the periventricular hypothalamus area of rats led to increased body weight, elevated food intake, and reduced locomotion.^28^*RELN* has been observed to be downregulated in the brains of SCZ patients, whereas a high-fat diet might result in elevated *RELN* proteins in the hypothalamus in mice.^29,30^

At the variant level, there are 3 shared variants (rs9868809, rs12107252, and rs12629759) with a CADD score >12.37 and a RegulomeDB score of 1f, supporting their deleteriousness and influences on gene expression, respectively.^31^ The 3 variants are eQTLs for *NCKIPSD* and *WDR6* in multiple brain regions. *NCKIPSD* was implicated by the genomic locus showing the strongest signal for AN in the latest GWAS and was involved in the maintenance of dendritic spines and synaptic activity.^12,32^*WDR6* was identified as a putative causal gene for AN by conditional analyses and probabilistic fine-mapping, and a rare variant in *WDR6* was found to be nominally associated with SCZ.^33,34^

Enrichment analysis revealed that the shared genes were highly involved in synapse organization. Abnormal brain development is widely acknowledged as one of the potential mechanisms underlying SCZ, and there is also preliminary evidence for neuroplasticity abnormalities in AN patients, supporting the pivotal role of synapse function in the onset of both disorders.^35,36^ It is noteworthy that SCZ-AN shared genes were also involved in metabolic processes such as the “hyaluronan catabolic process,” which is distinct from the neurological process dominant enrichment pattern identified for significant SCZ loci, supporting the view that AN has both psychiatric and metabolic underpinnings.^3^ Furthermore, despite the small number of genes included, genes mapped to the discordant shared loci were enriched in distinct biological processes compared with those mapped to the concordant loci, suggesting that they conferred divergent mechanisms.

As for the tissue-specific expression patterns, we found that the shared genes were overexpressed in several brain regions, including the pituitary and cerebellum. Since pathogeneses of both SCZ and AN involve dysfunction of metabolic and hypothalamic-pituitary-adrenal axis pathways, genetic enrichment in the pituitary is not surprising.^37,38^ Alteration of genetic expression in the cerebellum was previously reported for both SCZ and AN.^39,40^ Cerebellum might regulate feeding behaviors via projections to hypothalamic nuclei, and cognitive abnormality in SCZ was previously found to be associated with disruptions in the cortico-thalamic-cerebellar network.^41,42^ Moreover, 2 genes (*CELSR1* and *RELN*) located nearest to the shared loci identified by us showed particularly high expression in the cerebellum over multiple developmental time points (supplementary figure S6).

In the spatiotemporal genetic expression visualization, particularly high expression of shared genes was observed in the hippocampus in adolescence and the orbital frontal cortex in infancy (figure 4). The hippocampus is strongly implicated in higher-order decisions and can experience remarkable morphological development during adolescence. It was previously found to be implicated in the pathogenesis of SCZ and the modulation of contextually learned eating behaviors.^43–45^ The orbital frontal cortex is also involved in decision-making, and its thickness was identified as a state biomarker of AN.^46^ These indicate abnormal changes occurring in these brain regions may underlie the onset of SCZ and AN.

The current study has several limitations. First, despite the detected bidirectional causal effects, the implication of the causal relationship between SCZ and AN should be made with caution given the complex genetic architecture for psychiatric disorders and extensive pleiotropy across them. Second, since the recent GWAS for AN is much smaller than that for SCZ, our capability to detect shared loci is largely limited by the statistical power of AN GWAS. More shared loci are yet to be identified as the GWASs for both disorders grow larger. Third, the GWASs in this study are mainly based on populations of European ancestry, which limits the generalizability of our findings to other ancestry groups.^11,12^ Fourth, since GWASs can only capture the effects of common genetic variants, this study does not take into consideration the effects of rare variants and structural variations. Additionally, given the important role environmental factors play in the etiology of psychiatric disorders, future studies are required to explore their contribution to the co-occurrence of SCZ and AN. Finally, while this study only focuses on the genetic relationship between SCZ and AN, a GWAS for binge-eating disorder^47^ has now been published which will enable future studies to reveal shared genetic architecture between SCZ and a broader category of eating disorders.

In conclusion, this study confirms considerable polygenic overlap between SCZ and AN at the common genetic level and provides promising research targets for experimental studies by revealing shared genomic loci. The current study brings novel insights into the complex etiology underlying SCZ and AN and their genetic relationship. Future studies are called for to determine additional genomic loci underlying the shared genetic risk between SCZ and AN and elucidate how these loci influence the pathophysiology of the 2 disorders.

## Supplementary Material

Supplementary material is available at https://academic.oup.com/schizophreniabulletin/.

**Eating Disorders Working Group of the Psychiatric Genomics Consortium**

Roger Adan, Lars Alfredsson, Tetsuya Ando, Ole Andreassen, Jessica Baker, Andrew Bergen, Wade Berrettini, Andreas Birgegård, Joseph Boden, Ilka Boehm, Vesna Boraska Perica, Harry Brandt, Gerome Breen, Julien Bryois, Katharina Buehren, Cynthia Bulik, Roland Burghardt, Matteo Cassina, Sven Cichon, Jonathan Coleman, Roger Cone, Philippe Courtet, Steven Crawford, Scott Crow, James Crowley, Unna Danner, Oliver Davis, Martina de Zwaan, George Dedoussis, Janiece DeSocio, Danielle Dick, Dimitris Dikeos, Christian Dina, Monika Dmitrzak-Weglarz, Elisa Docampo, Laramie Duncan, Karin Egberts, Stefan Ehrlich, Geòrgia Escaramís, Tõnu Esko, Xavier Estivill, Anne Farmer, Angela Favaro, Fernando Fernández-Aranda, Krista Fischer, Manuel Föcker, Lenka Foretova, Andreas Forstner, Monica Forzan, Christopher Franklin, Steven Gallinger, Ina Giegling, Paola Giusti-Rodríguez, Fragiskos Gonidakis, Scott Gordon, Philip Gorwood, Monica Gratacos Mayora, Jakob Grove, Sébastien Guillaume, Yiran Guo, Hakon Hakonarson, Katherine Halmi, Ken Hanscombe, Konstantinos Hatzikotoulas, Joanna Hauser, Johannes Hebebrand, Sietske Helder, Stefan Herms, Beate Herpertz-Dahlmann, Wolfgang Herzog, Anke Hinney, L. John Horwood, Christopher Hübel, Laura Huckins, James Hudson, Hartmut Imgart, Hidetoshi Inoko, Vladimir Janout, Susana Jiménez-Murcia, Craig Johnson, Jennifer Jordan, Antonio Julià, Gursharan Kalsi, Deborah Kaminská, Allan Kaplan, Jaakko Kaprio, Leila Karhunen, Andreas Karwautz, Martien Kas, Walter Kaye, James Kennedy, Martin Kennedy, Anna Keski-Rahkonen, Kirsty Kiezebrink, Youl-Ri Kim, Lars Klareskog, Kelly Klump, Mikael Landén, Janne Larsen, Stephanie Le Hellard, Virpi Leppä, Dong Li, Paul Lichtenstein, Lisa Lilenfeld, Bochao Danae Lin, Jolanta Lissowska, Jurjen Luykx, Mario Maj, Sara Marsal, Nicholas Martin, Manuel Mattheisen, Morten Mattingsdal, Sarah Medland, Andres Metspalu, Ingrid Meulenbelt, Nadia Micali, Karen Mitchell, James Mitchell, Alessio Maria Monteleone, Palmiero Monteleone, Preben Bo Mortensen, Melissa Munn-Chernoff, Benedetta Nacmias, Marie Navratilova, Ioanna Ntalla, Catherine Olsen, Roel Ophoff, Leonid Padyukov, Jacques Pantel, Hana Papezova, Richard Parker, John Pearson, Nancy Pedersen, Liselotte Petersen, Dalila Pinto, Kirstin Purves, Anu Raevuori, Nicolas Ramoz, Ted Reichborn-Kjennerud, Valdo Ricca, Samuli Ripatti, Stephan Ripke, Franziska Ritschel, Marion Roberts, Dan Rujescu, Filip Rybakowski, Paolo Santonastaso, André Scherag, Stephen Scherer, Ulrike Schmidt, Nicholas Schork, Alexandra Schosser, Jochen Seitz, Lenka Slachtova, P. Eline Slagboom, Margarita Slof-Op 't Landt, Agnieszka Slopien, Sandro Sorbi, Michael Strober, Patrick Sullivan, Beata Świątkowska, Jin Szatkiewicz, Elena Tenconi, Laura Thornton, Alfonso Tortorella, Janet Treasure, Artemis Tsitsika, Marta Tyszkiewicz-Nwafor, Annemarie van Elburg, Eric van Furth, Tracey Wade, Gudrun Wagner, Hunna Watson, Thomas Werge, David Whiteman, Elisabeth Widen, D. Blake Woodside, Shuyang Yao, Zeynep Yilmaz, Eleftheria Zeggini, Stephanie Zerwas, Stephan Zipfel. Co-Chairs: Gerome Breen, Cynthia Bulik.

sbae087_suppl_Supplementary_Material
