# Supplementary material for: Shared Genetic Architecture Between Schizophrenia and Anorexia Nervosa: A Cross-trait Genome-Wide Analysis
Source: Schizophr Bull. 2024 Jun 7;50(5):1255–65. doi: 10.1093/schbul/sbae087 (PMC11349005; doi:10.1093/schbul/sbae087)

Supplementary material: Shared genetic architecture between schizophrenia and anorexia nervosa: a cross-trait genome-wide analysis, Lu et al.

Eating Disorders Working Group of the Psychiatric Genomics Consortium

Supplementary Methods

Supplementary Results

**Supplementary table 1. F statistics of instrumental variables for SCZ and AN**

**Supplementary table 2. Results for bidrectional Mendelian randomization analysis for SCZ and AN**

**Supplementary table 3. Heterogeneity and horizontal pleiotropy analyses between SCZ and AN**

**Supplementary table 4. Leave-one-out analyses for instrumental variables.**

**Supplementary table 5. Results for MiXeR analysis of SCZ and AN**

**Supplementary table 6. Results for MiXeR analysis of AN and height, obsessive compulsive disorder (OCD) and anxiety disorder(ANX)**

**Supplementary table 7. Novel genomic loci associated with SCZ conditional on the association with AN at condFDR < 0.01**

**Supplementary table 8. Novel genomic loci associated with AN conditional on the association with SCZ at condFDR < 0.01**

**Supplementary Table 9. Novel genomic loci jointly associated with SCZ and AN at conjFDR < 0.1**

**Supplementary Table 10. All candidate SNPs in genomic loci jointly associated with SCZ and AN having a conjFDR <0.05 and an r2<0.6 with one of the independent significant SNPs**

**Supplementary Table 11. All candidate SNPs in genomic loci jointly associated with SCZ and AN having a conjFDR <0.1 and an r2<0.6 with one of the independent significant SNPs**

**Supplementary Table 12. Genes mapped to the genomic loci jointly associated with SCZ and AN**

**Supplementary Table 13. GO terms significantly associated with genes mapped to the shared loci at conjFDR < 0.05**

**Supplementary Table 14. GO terms significantly associated with genes mapped to the significant loci for SCZ conditional on AN at condFDR < 0.01**

**Supplementary Table 15. Magma tissue expression analysis of genes mapped to the shared loci at conjFDR < 0.05**

**Supplementary Table 16. Genes mapped to the genomic loci jointly associated with SCZ and AN at conjFDR < 0.1**

**Supplementary Table 17. GO terms significantly associated with genes mapped to the three disordant shared loci at conjFDR < 0.1**

**Supplementary figure 1. Funnel plots of two-sample Mendelian randomization analysis of SCZ and AN**

**Supplementary figure 2. Polygenic overlap between anorexia nervosa (AN) and height, obsessive compulsive disorder (OCD) and anxiety disorder (ANX)**

**Supplementary figure 3. The conditional quantile-quantile(Q-Q) plot and negative log-likelihood plot generated from bivariate MiXeR analysis
Supplementary figure 4. Gene Ontology (GO) analysis of genes mapped to the shared loci between SCZ and AN**

**Supplementary figure 5. Proportion of concordant variants as a function of the conjunctional FDR thresholds**

**Supplementary figure 6. Spatiotemporal gene expression pattern of 6 genes nearest to the shared loci between SCZ and AN across 16 brain regions and 11 developmental time points**

**Eating Disorders Working Group of the Psychiatric Genomics Consortium**

Roger Adan, Lars Alfredsson, Tetsuya Ando, Ole Andreassen, Jessica Baker, Andrew Bergen, Wade Berrettini, Andreas Birgegård, Joseph Boden, Ilka Boehm, Vesna Boraska Perica, Harry Brandt, Gerome Breen, Julien Bryois, Katharina Buehren, Cynthia Bulik, Roland Burghardt, Matteo Cassina, Sven Cichon, Jonathan Coleman, Roger Cone, Philippe Courtet, Steven Crawford, Scott Crow, James Crowley, Unna Danner, Oliver Davis, Martina de Zwaan, George Dedoussis, Janiece DeSocio, Danielle Dick, Dimitris Dikeos, Christian Dina, Monika Dmitrzak-Weglarz, Elisa Docampo, Laramie Duncan, Karin Egberts, Stefan Ehrlich, Geòrgia Escaramís, Tõnu Esko, Xavier Estivill, Anne Farmer, Angela Favaro, Fernando Fernández-Aranda, Krista Fischer, Manuel Föcker, Lenka Foretova, Andreas Forstner, Monica Forzan, Christopher Franklin, Steven Gallinger, Ina Giegling, Paola Giusti-Rodríguez, Fragiskos Gonidakis, Scott Gordon, Philip Gorwood, Monica Gratacos Mayora, Jakob Grove, Sébastien Guillaume, Yiran Guo, Hakon Hakonarson, Katherine Halmi, Ken Hanscombe, Konstantinos Hatzikotoulas, Joanna Hauser, Johannes Hebebrand, Sietske Helder, Stefan Herms, Beate Herpertz-Dahlmann, Wolfgang Herzog, Anke Hinney, L. John Horwood, Christopher Hübel, Laura Huckins, James Hudson, Hartmut Imgart, Hidetoshi Inoko, Vladimir Janout, Susana Jiménez-Murcia, Craig Johnson, Jennifer Jordan, Antonio Julià, Gursharan Kalsi, Deborah Kaminská, Allan Kaplan, Jaakko Kaprio, Leila Karhunen, Andreas Karwautz, Martien Kas, Walter Kaye, James Kennedy, Martin Kennedy, Anna Keski-Rahkonen, Kirsty Kiezebrink, Youl-Ri Kim, Lars Klareskog, Kelly Klump, Mikael Landén, Janne Larsen, Stephanie Le Hellard, Virpi Leppä, Dong Li, Paul Lichtenstein, Lisa Lilenfeld, Bochao Danae Lin, Jolanta Lissowska, Jurjen Luykx, Mario Maj, Sara Marsal, Nicholas Martin, Manuel Mattheisen, Morten Mattingsdal, Sarah Medland, Andres Metspalu, Ingrid Meulenbelt, Nadia Micali, Karen Mitchell, James Mitchell, Alessio Maria Monteleone, Palmiero Monteleone, Preben Bo Mortensen, Melissa Munn-Chernoff, Benedetta Nacmias, Marie Navratilova, Ioanna Ntalla, Catherine Olsen, Roel Ophoff, Leonid Padyukov, Jacques Pantel, Hana Papezova, Richard Parker, John Pearson, Nancy Pedersen, Liselotte Petersen, Dalila Pinto, Kirstin Purves, Anu Raevuori, Nicolas Ramoz, Ted Reichborn-Kjennerud, Valdo Ricca, Samuli Ripatti, Stephan Ripke, Franziska Ritschel, Marion Roberts, Dan Rujescu, Filip Rybakowski, Paolo Santonastaso, André Scherag, Stephen Scherer, Ulrike Schmidt, Nicholas Schork, Alexandra Schosser, Jochen Seitz, Lenka Slachtova, P. Eline Slagboom, Margarita Slof-Op 't Landt, Agnieszka Slopien, Sandro Sorbi, Michael Strober, Patrick Sullivan, Beata Świątkowska, Jin Szatkiewicz, Elena Tenconi, Laura Thornton, Alfonso Tortorella, Janet Treasure, Artemis Tsitsika, Marta Tyszkiewicz-Nwafor, Annemarie van Elburg, Eric van Furth, Tracey Wade, Gudrun Wagner, Hunna Watson, Thomas Werge, David Whiteman, Elisabeth Widen, D. Blake Woodside, Shuyang Yao, Zeynep Yilmaz, Eleftheria Zeggini, Stephanie Zerwas, Stephan Zipfel. Co-Chairs: Gerome Breen, Cynthia Bulik .

Supplementary Methods

*Two-sample Mendelian randomization (MR) Analysis*

To evaluate the causal effect of genetic predisposition of SCZ on AN, we performed a two-sample MR analysis, using SCZ GWAS as the exposure dataset and AN GWAS as the outcome dataset (1, 2). First, SNPs passing the significance threshold (p-value < 5 × 10^-8^) were extracted from SCZ GWAS as instrumental variables. The instrumental variables were then clumped based on the 1000 Genomes Project Phase 3 panel. Only the index SNPs (SNPs in approximate linkage equilibrium at r^2^ < 0.001 with any other associated SNP within 10MB) with the minimum p-value were retained for subsequent analyses. Next, the exposure and outcome data were harmonized to ensure the concordance of effect alleles between datasets. SNPs with non-concordant alleles and palindromic SNPs were excluded from instrumental variables.

To detect potential weak instrumental variables, we calculated the F statistic for each instrumental variable, which was defined as F = R^2^× (n − k − 1)/k× (1 − R^2^) (R^2^, variance of exposure explained by an instrumental variable, acquired from MR Steiger directionality test; n, sample size; and k, number of instrumental variables) (3). Variants with F statistic < 10 were excluded from instrumental variables (4).

After that, a Wald ratio estimate was calculated for each variant, and random-effects inverse variance weighted meta-analysis (IVW-mre) was employed to summarize the effects across all instrumental variables (5). The weighted median (WME), weighted mode (WMO), Mendelian randomization-Egger (MR-Egger) methods were employed in complementary analyses, as these methods generate results based on different assumptions on the validity of instrument variables (6, 7, 8).

Furthermore, sensitivity analyses were conducted to check whether there were potential violations of the MR model assumptions. Cochran’s Q test for the random effects inverse variance weighted method was performed to evaluate the heterogeneity in causal effects across instrumental variables. MR-Egger regression was conducted to check horizontal pleiotropy of instrumental variables (9). The Mendelian randomization pleiotropy residual sum and outlier (MR-PRESSO) test was performed to detect horizontal pleiotropic outliers among instrumental variables and provide effect estimates after outlier correction (9).

We also tested for the causal influences of genetic predisposition of AN on SCZ, using the same procedures described above. The only difference was that we adopted a more relaxed p-value threshold (p-value < 1 × 10^-6^) to select instrumental variables from AN GWAS, given that no SNP was retained after clumping at the genome-wide significance level. All statistical analyses were performed using the R package TwoSampleMR (“0.5.6”) and MRPRESSO (“1.0”).

*MiXeR analysis*

To quantify polygenic overlap between SCZ and AN, we applied a causal mixture model to GWAS summary statistics, using MiXeR v1.3. MiXeR can estimate the number of trait-influencing variants with non-zero effects required to explain 90% of the disorder’s heritability as well as the number of shared variants between disorders irrespective of the direction of effect.

A univariate MiXeR model was first constructed for SCZ and AN respectively to estimate their polygenicity (proportion of non-null SNPs) and discoverability (variance of effect sizes of non-null SNPs). Univariate estimates and standard deviations for SCZ and AN were calculated by performing 20 iterations with 2 million randomly selected SNPs in 1000 Genomes Phase 3 data, followed by random pruning at an LD threshold of r^2^=0.8(10). The mean and standard deviation were then computed for each variable from the resulting sample of 20 iterations for each analysis.

A bivariate MiXeR model was then constructed to divide the genetic effects for two disorders into four components: (1) SNPs associated with both disorders, (2) SNPs associated with neither of the two disorders , and (3+4) one component each for SNPs specific to each disorder ; the estimated number of variants in each component is visualized as a Venn diagram (11). Genetic association and polygenic overlap are summarized as genetic correlation and Dice Coefficient (DC, estimated proportion of shared variants out of all variants with non-zero effects), respectively (11). The model fit can be evaluated by the predicted versus observed conditional quantile–quantile (Q-Q) plots and Akaike Information Criterion (AIC) (11).

In order to offer references for polygenic overlap between SCZ and AN, we also conducted MiXeR analysis for AN vs. height, AN vs. obsessive compulsive disorder and AN vs. anxiety disorder using the same procedures described above.

MiXeR software and codes are available online at <https://github.com/precimed/mixer>. More technical details about MiXeR are available in Frei et al.(11).

*Cond/conjFDR analysis*

We applied conditional FDR (condFDR) for genomic locus detection. CondFDR is an extension of the conventional false discover rate in an empirical Bayesian framework, allowing inference on genetic variants associated with a primary disorder conditional on their genetic association with a secondary disorder (12). It is defined as the probability that a SNP has a null association with the primary disorder conditional on the fact that the p-value for the secondary disorder is as small or smaller than the observed p-value. This approach can substantially boost statistical power for genomic locus discovery by combining the power of two GWASs and is frequently utilized to discover novel risk loci for a series of disorders (13, 14, 15, 16).

We used conjunctive FDR (conjFDR), an extension of condFDR to identify shared genomic loci between SCZ and AN. ConjFDR is defined as the maximum of the two condFDR values produced by exchanging the roles of primary and secondary disorder in condFDR analysis, and provides a conservative FDR estimate for a genetic variant to be jointly associated with both disorders, regardless of whether their effects are concordant or discordant.

To ensure independence of genetic variants involved, linkage disequilibrium-based random pruning was performed at r^2^ < 0.05 based on 1,000 independent iterations before cond/conjFDR estimation. Genetic variants around the major histocompatibility complex (MHC) (chr6:25119106–33854733, hg19) and chromosome 8 inversion (chr8:7200000–12500000, hg19) were excluded from this estimation step due to their intricate linkage disequilibrium, and their cond/conjFDR values were imputed afterwards via post-hoc estimation.

Significance thresholds were set at condFDR < 0.01 and conjFDR < 0.05 as recommended (17, 18). Given the limited power of AN GWAS, we also used a more relaxed threshold of conjFDR < 0.1 to boost loci discovery. All cond/conjFDR statistical analyses were performed using the R package cfdr.pleio ("0.0.0.9100") available online at <https://github.com/alexploner/cfdr.pleio>.

*Definition of genomic loci, functional annotation, gene mapping, and expression analysis*

We defined the genomic loci following the Functional Mapping and Annotation (FUMA) protocol recommendations (19). Independent significant SNPs were defined as SNPs independent with each other at r^2^ < 0.6 and with condFDR<0.01 or conjFDR<0.05/conjFDR < 0.1, respectively. A subgroup of independent significant SNPs in approximate linkage equilibrium at r^2^ < 0.1 were selected as lead SNPs. Physically overlapping genomic loci were merged as one locus (<250 kb apart), and the SNP with lowest cond/conjFDR was selected as lead SNP for the locus. Candidate SNPs in each locus were defined as those in linkage disequilibrium at r^2^ >0.6 with any of the independent significant SNPs, and having a condFDR<0.01 or conjFDR<0.05/conjFDR < 0.1. All LD information was calculated using the European subset of 1000 Genomes Project Phase 3 reference panel (20). We determined whether the effect directions for SCZ and AN were concordant or discordant by contrasting the beta values of the lead SNP in the shared loci for the two disorders. To define novel loci, we compared the loci identified by cond/conjFDR analyses with the original and subsequent GWAS summary statistics for SCZ and AN(1, 2, 13, 18, 21, 22) , using a publicly available script from at <https://github.com/precimed/yunhoop>

FUMA v1.5.0 (<https://fuma.ctglab.nl/>) was utilized to functionally annotate all candidate SNPs in linkage disequilibrium at r^2^ >0.6 with one of the independent significant SNPs in cond/conjFDR analyses (19). Four strategies were employed to annotate SNPs: (1) ANNOVAR to annotate the functional consequences of genetic variants; (2) combined annotation-dependent depletion (CADD) scores to predict how deleterious the effects of genetic variants are on protein structures, with scores > 12.37 indicating deleteriousness (23); (3) RegulomeDB scores to predict the probability of regulatory functions of genetic variants, with lower scores indicating higher likelihood of regulatory functions (24); (4) chromatin states to predict the transcriptional and regulatory effects using 15 categorical states across 127 tissues, with scores from 1 to 7 indicating open chromatin states vulnerable to regulatory elements (25, 26).

To identify shared genes, we employed three strategies to map genes to candidate SNPs in shared loci via FUMA v1.5.0 (19): (1) positional mapping, matching SNPs to genes based on physical proximity (within 10kb); (2) expression quantitative trait locus (eQTL) mapping, linking genes to cis-eQTL SNPs whose variations are associated with gene expression level, based on the GTEx v8 and Braineac databases (27, 28); (3) chromatin interaction maping: mapping of SNPs to genes based on three-dimensional DNA-DNA interactions between SNPs and genes. Genes identified by at least one of the mapping strategies were defined as shared genes. By querying the GTEx v8 database, we performed MAGMA tissue expression analysis via FUMA v1.5.0 to explore whether shared genes were expressed in specific tissues (19, 27).

To explore whether genes were significantly enriched in specific biological pathways, we applied gene ontology (GO) analysis to all genes mapped to the shared loci at conjFDR < 0.05 as well as the three discordant shared genomic loci at conjFDR < 0.1 (29). GO analysis was performed with the R package clusterProfiler (“3.18.1”), and the results were visualized using the R package enrichplot (“1.10.2”).

To visualize spatiotemporal genetic expression pattern, we generated the heatmaps illustrating the expression differences between shared genes versus 52,376 background genes with non-zero expression across 16 brain regions and 11 developmental time points based on BrainSpan RNA sequencing data (30, 31). BrainSpan captures the genetic expression levels in different brain regions from 8 postconceptual weeks (pcw) to 40 years. The specific time points used in this study were 16 pcw (mid fetal), 37 pcw (late fetal), 4 months (newborn), 1 year (infancy), 3 years (early childhood), 8 years (middle childhood), 13 years (late childhood), 19 years (adolescence), 21 years (late adolescence), 30 years (early adulthood), and 36 years (middle adulthood) (32). First, we log2-transformed genetic expression levels. Next, we subtracted mean transformed expression value of all background genes from mean transformed expression value of shared genes in each brain region at each time point. Before generating the heatmap, we centered and scaled the expression differences by developmental time point, so that the expression differences were comparable across brain regions at each specific time point. The heatmap was generated using the R function “heatmap”. We generated heatmap both for the average expression of 127 shared genes available in BrainSpan and 6 specific shared genes (CELSR1, NEGR1, RELN, NCAM1, SOX5 and TSNARE1) located nearest to shared loci.

**Supplementary results**

*Two-sample mendelian randomization analysis*

A total of 127 and 18 SNPs were selected as instrumental variables for SCZ and AN, respectively. The F statistics ranged from 15.7 to 39.0 (median = 18.0, interquartile range = 4.1) for instrumental variables of SCZ, while the statistics ranged from 71.2 to 89.1 (median = 78.8, interquartile range = 6.5) for instrumental variables of AN (Supplementary table 1). The genetic predisposition to SCZ was found to be positively associated with AN using the IVW-mre method, WME method and WMO method (Odds ratio for IVW-mre = 1.13, 95% confidence interval 1.06-1.19, p-value = 3.84 × 10^-5^; odds ratio for WME = 1.15, 95% confidence interval 1.08-1.23, p-value = 3.53 × 10^-5^; odds ratio for WMO = 1.19, 95% confidence interval 1.00-1.42, p-value = 0.046), as indicated in table S2. Genetically predicted AN was found to be positively associated with SCZ using the IVW-mre method (Odds ratio = 1.15, 95% confidence interval, 1.05-1.27, p-value = 0.003), but not with the other three methods (Supplementary table 2). These results suggest an inconclusive bidirectional causal effect between SCZ and AN (figure 1).

Cochran’s Q test detected heterogeneity of effects across instrumental variables and supported the employment of random-effect methods (Supplementary table 3). MR-Egger regression analysis did not detect horizontal pleiotropy of instrumental variables since the intercept of MR-Egger did not significantly deviate from zero (Supplementary table 3). Funnel plots did not detect directional pleiotropy, as causal estimates from weaker instrumental variables were not skewed in one direction (Supplementary figure 1). MR-PRESSO test detected two outliers for SCZ on AN and one outlier for AN on SCZ, and confirmed their bidirectional causal relationship by outlier correction (Odds ratio of SCZ on AN = 1.11, 95% confidence interval, 1.05-1.17, p-value = 2.68 × 10^-4^; odds ratio of AN on SCZ = 1.13, 95% confidence interval, 1.03-1.23, p-value = 0.02) (Supplementary table 2). Leave-one-out analyses suggested the causal effect was not driven by a single instrumental variable (Supplementary table 4).

**Supplementary References**

1. Trubetskoy V, Pardiñas AF, Qi T, Panagiotaropoulou G, Awasthi S, Bigdeli TB, et al. Mapping genomic loci implicates genes and synaptic biology in schizophrenia. Nature. 2022;604(7906):502-8.

2. Watson HJ, Yilmaz Z, Thornton LM, Hübel C, Coleman JRI, Gaspar HA, et al. Genome-wide association study identifies eight risk loci and implicates metabo-psychiatric origins for anorexia nervosa. Nat Genet. 2019;51(8):1207-14.

3. Pierce BL, Ahsan H, Vanderweele TJ. Power and instrument strength requirements for Mendelian randomization studies using multiple genetic variants. Int J Epidemiol. 2011;40(3):740-52.

4. Staiger DO, Stock JH. Instrumental variables regression with weak instruments. National Bureau of Economic Research Cambridge, Mass., USA; 1994.

5. Lawlor DA, Harbord RM, Sterne JA, Timpson N, Davey Smith G. Mendelian randomization: using genes as instruments for making causal inferences in epidemiology. Stat Med. 2008;27(8):1133-63.

6. Bowden J, Davey Smith G, Haycock PC, Burgess S. Consistent Estimation in Mendelian Randomization with Some Invalid Instruments Using a Weighted Median Estimator. Genet Epidemiol. 2016;40(4):304-14.

7. Hartwig FP, Davey Smith G, Bowden J. Robust inference in summary data Mendelian randomization via the zero modal pleiotropy assumption. Int J Epidemiol. 2017;46(6):1985-98.

8. Bowden J, Davey Smith G, Burgess S. Mendelian randomization with invalid instruments: effect estimation and bias detection through Egger regression. Int J Epidemiol. 2015;44(2):512-25.

9. Verbanck M, Chen CY, Neale B, Do R. Detection of widespread horizontal pleiotropy in causal relationships inferred from Mendelian randomization between complex traits and diseases. Nat Genet. 2018;50(5):693-8.

10. Bulik-Sullivan BK, Loh PR, Finucane HK, Ripke S, Yang J, Patterson N, et al. LD Score regression distinguishes confounding from polygenicity in genome-wide association studies. Nat Genet. 2015;47(3):291-5.

11. Frei O, Holland D, Smeland OB, Shadrin AA, Fan CC, Maeland S, et al. Bivariate causal mixture model quantifies polygenic overlap between complex traits beyond genetic correlation. Nat Commun. 2019;10(1):2417.

12. Smeland OB, Frei O, Shadrin A, O'Connell K, Fan CC, Bahrami S, et al. Discovery of shared genomic loci using the conditional false discovery rate approach. Hum Genet. 2020;139(1):85-94.

13. Smeland OB, Shadrin A, Bahrami S, Broce I, Tesli M, Frei O, et al. Genome-wide Association Analysis of Parkinson's Disease and Schizophrenia Reveals Shared Genetic Architecture and Identifies Novel Risk Loci. Biol Psychiatry. 2021;89(3):227-35.

14. Rødevand L, Bahrami S, Frei O, Lin A, Gani O, Shadrin A, et al. Polygenic overlap and shared genetic loci between loneliness, severe mental disorders, and cardiovascular disease risk factors suggest shared molecular mechanisms. Transl Psychiatry. 2021;11(1):3.

15. Bahrami S, Shadrin A, Frei O, O'Connell KS, Bettella F, Krull F, et al. Genetic loci shared between major depression and intelligence with mixed directions of effect. Nat Hum Behav. 2021;5(6):795-801.

16. Drange OK, Smeland OB, Shadrin AA, Finseth PI, Witoelar A, Frei O, et al. Genetic Overlap Between Alzheimer's Disease and Bipolar Disorder Implicates the MARK2 and VAC14 Genes. Front Neurosci. 2019;13:220.

17. Andreassen OA, Thompson WK, Schork AJ, Ripke S, Mattingsdal M, Kelsoe JR, et al. Improved detection of common variants associated with schizophrenia and bipolar disorder using pleiotropy-informed conditional false discovery rate. PLoS Genet. 2013;9(4):e1003455.

18. Smeland OB, Bahrami S, Frei O, Shadrin A, O'Connell K, Savage J, et al. Genome-wide analysis reveals extensive genetic overlap between schizophrenia, bipolar disorder, and intelligence. Mol Psychiatry. 2020;25(4):844-53.

19. Watanabe K, Taskesen E, van Bochoven A, Posthuma D. Functional mapping and annotation of genetic associations with FUMA. Nat Commun. 2017;8(1):1826.

20. Auton A, Brooks LD, Durbin RM, Garrison EP, Kang HM, Korbel JO, et al. A global reference for human genetic variation. Nature. 2015;526(7571):68-74.

21. Ahangari M, Everest E, Nguyen TH, Verrelli BC, Webb BT, Bacanu SA, et al. Genome-wide analysis of schizophrenia and multiple sclerosis identifies shared genomic loci with mixed direction of effects. Brain Behav Immun. 2022;104:183-90.

22. Johnson EC, Kapoor M, Hatoum AS, Zhou H, Polimanti R, Wendt FR, et al. Investigation of convergent and divergent genetic influences underlying schizophrenia and alcohol use disorder. Psychol Med. 2023;53(4):1196-204.

23. Kircher M, Witten DM, Jain P, O'Roak BJ, Cooper GM, Shendure J. A general framework for estimating the relative pathogenicity of human genetic variants. Nat Genet. 2014;46(3):310-5.

24. Boyle AP, Hong EL, Hariharan M, Cheng Y, Schaub MA, Kasowski M, et al. Annotation of functional variation in personal genomes using RegulomeDB. Genome Res. 2012;22(9):1790-7.

25. Kundaje A, Meuleman W, Ernst J, Bilenky M, Yen A, Heravi-Moussavi A, et al. Integrative analysis of 111 reference human epigenomes. Nature. 2015;518(7539):317-30.

26. Zhu Z, Zhang F, Hu H, Bakshi A, Robinson MR, Powell JE, et al. Integration of summary data from GWAS and eQTL studies predicts complex trait gene targets. Nat Genet. 2016;48(5):481-7.

27. Battle A, Brown CD, Engelhardt BE, Montgomery SB. Genetic effects on gene expression across human tissues. Nature. 2017;550(7675):204-13.

28. Ramasamy A, Trabzuni D, Guelfi S, Varghese V, Smith C, Walker R, et al. Genetic variability in the regulation of gene expression in ten regions of the human brain. Nat Neurosci. 2014;17(10):1418-28.

29. Ashburner M, Ball CA, Blake JA, Botstein D, Butler H, Cherry JM, et al. Gene ontology: tool for the unification of biology. The Gene Ontology Consortium. Nat Genet. 2000;25(1):25-9.

30. Miller JA, Ding SL, Sunkin SM, Smith KA, Ng L, Szafer A, et al. Transcriptional landscape of the prenatal human brain. Nature. 2014;508(7495):199-206.

31. BrainSpan. BrainSpan atlas of the developing human brain. Published 2010. Accessed 15 May 2023. http://www.brainspan.org/

32. Bahrami S, Hindley G, Winsvold BS, O’Connell KS, Frei O, Shadrin A, et al. Dissecting the shared genetic basis of migraine and mental disorders using novel statistical tools. Brain. 2022;145(1):142-53.

**Supplementary figure 1. Funnel plots of two-sample Mendelian randomization analysis of SCZ and AN**

Scatterplot of inverse standard errors on the y-axis versus estimated beta (log-OR) for the target trait on the x-axis, for all instrumental variables selected for the causal trait. The vertical line represents the overall causal effect estimated by the random-effects inverse variance weighted method.

(A): causal trait = SCZ, target trait = AN, n = 127;

(B): causal trait = AN, target trait = SCZ, n = 18.


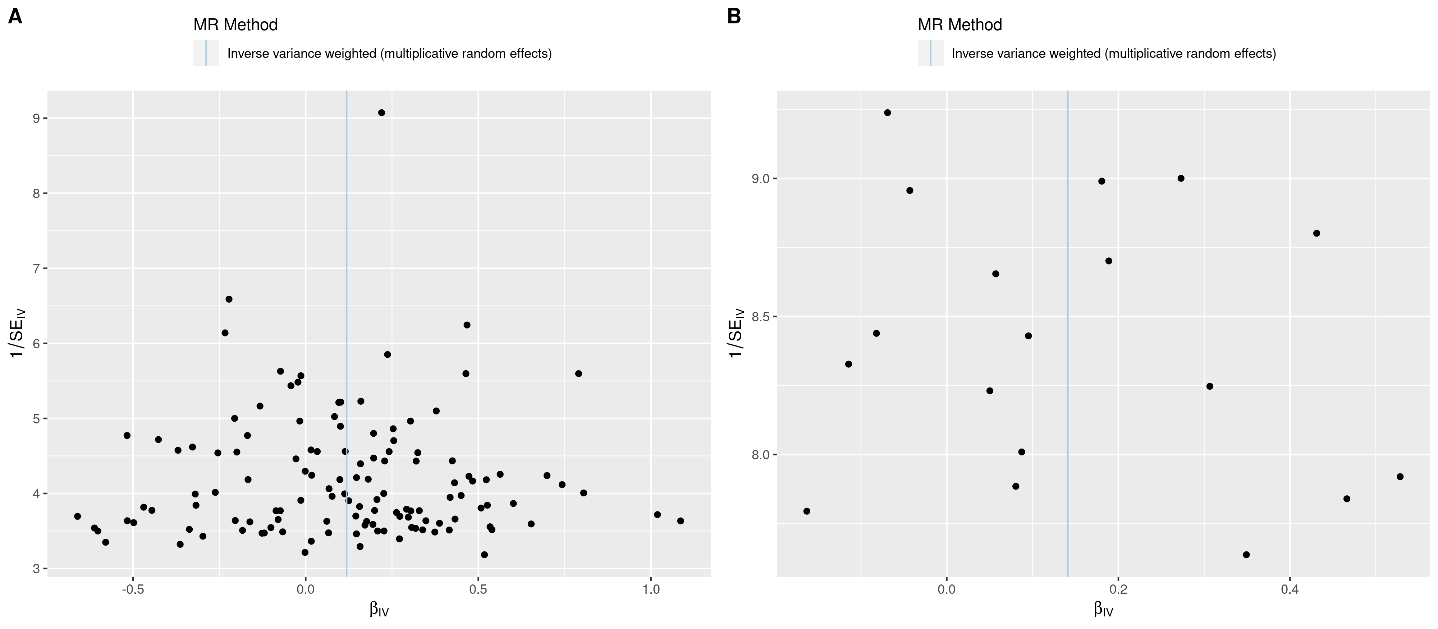


**Supplementary figure 2. Polygenic overlap between anorexia nervosa (AN) and height, obsessive compulsive disorder (OCD) and anxiety disorder (ANX)**

The Venn diagrams illustrating the estimated number of non-null variants shared between and specific to each disorder for AN vs. height (A), AN vs. OCD (B) and AN vs. ANX (C) : numbers in the circle indicate quantity (standard error) of genetic variants in thousands. The blue circle represents AN, the grey part represents overlap and the orange circle represents height in (A), OCD in (B) and ANX in (C). The size of the circle indicates polygenicity, with larger circle reflecting greater polygenicity. The estimated genetic correlation is also shown below the Venn diagram, with an accompanying directional scale (red indicates positive correlation).


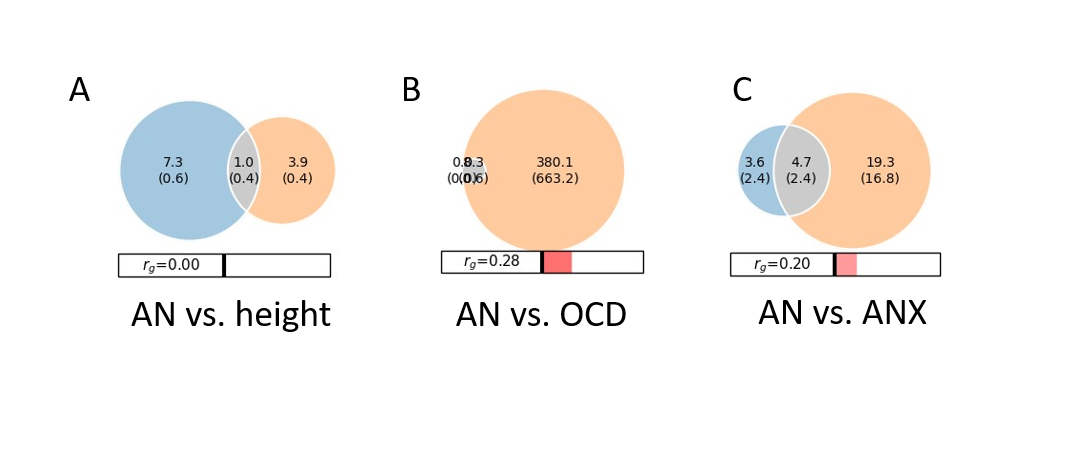


**Supplementary figure 3. The conditional quantile-quantile(Q-Q) plot and negative log-likelihood plot generated from bivariate MiXeR analysis**(A) and (B) show conditional Q-Q plots of observed and expected -log10 p-values for the primary trait1 vs the expected -log10 p-values under the global null hypothesis, both for the full data set and conditioned on the p-values for the secondary trait2 at levels p ≤ 0.1, p ≤ 0.01 and p ≤ 0.001, indicated by color, where solid lines indicate observed quantiles and dashed lines indicate model predictions in each stratum. The black dashed line is the expected Q-Q plot under the null hypothesis of no association with the primary trait. In (A): primary trait1 = SCZ, conditioning trait2 = AN; In (B): primary trait2 = AN, conditioning trait1 = SCZ.
(C) shows the negative log-likelihood of the bivariate MiXeR fit as a function of extent of polygenic overlap between SCZ and AN.


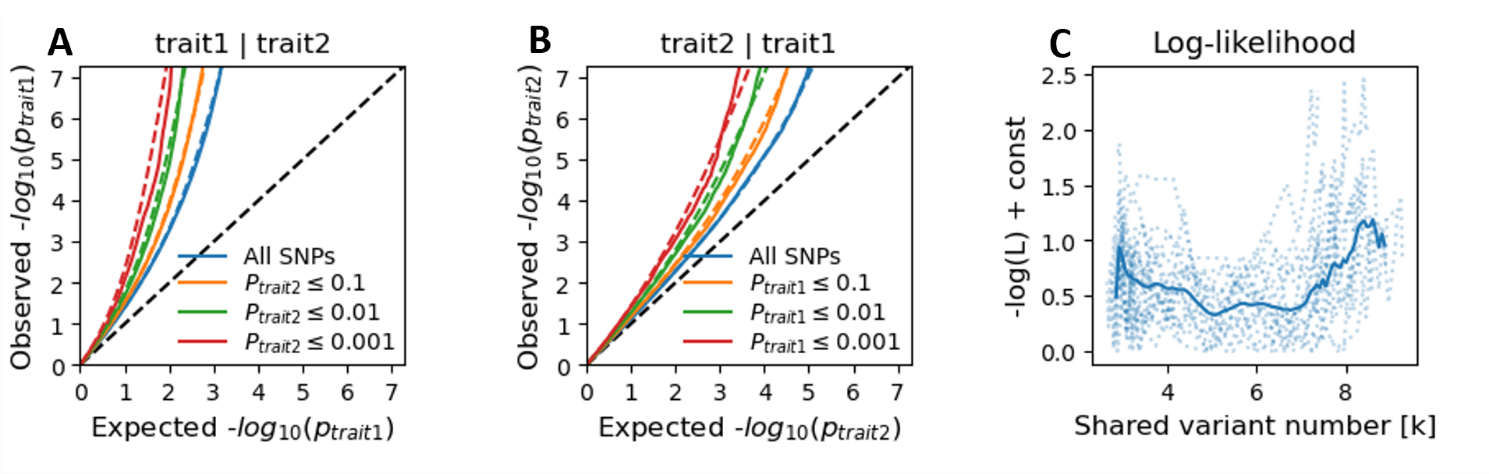


**Supplementary figure 4. Gene Ontology (GO) analysis of genes mapped to the shared loci between SCZ and AN**
(A) visualizes the top five GO categories associated with the shared genes (n = 130). Each GO category is represented by a yellow point, with its size indicating the number of enriched genes and radial lines pointing to the names of enriched genes, with the different GO categories represented by line color.
(B) shows the correlation network of the 12 GO categories significantly associated with the shared genes at Benjamini-Hochberg FDR < 0.05. Each GO category is represented by one point, with its size indicating the number of enriched genes and color indicating the FDR.


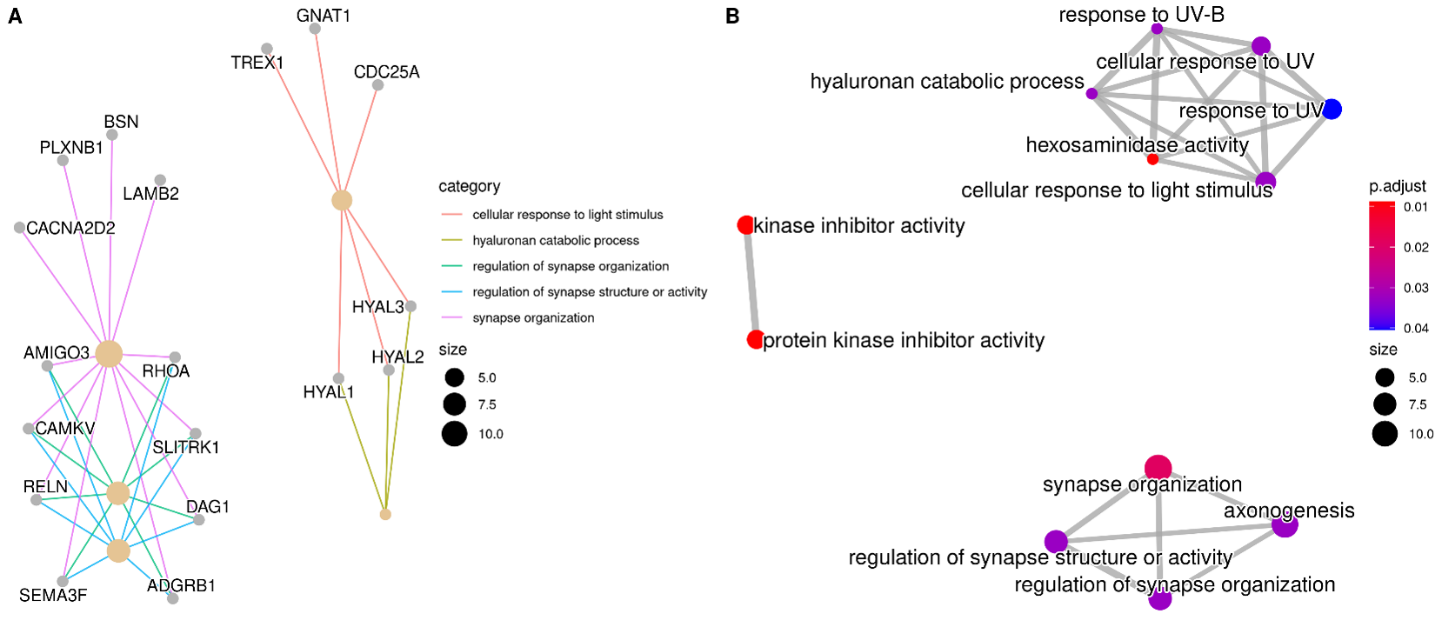


**Supplementary figure 5. Proportion of concordant variants as a function of the conjunctional FDR thresholds**

The plot shows the proportion of concordant variants (on the vertical axis) as a function of the conjunctional FDR threshold (on the horizontal axis): every point on the curve indicates the proportion of concordance for all variants that fall below the indicated threshold (to the right of the threshold, as the horizontal axis is sorted from least to most significant). The upper tick marks on the horizontal axis indicate inidividual variants.


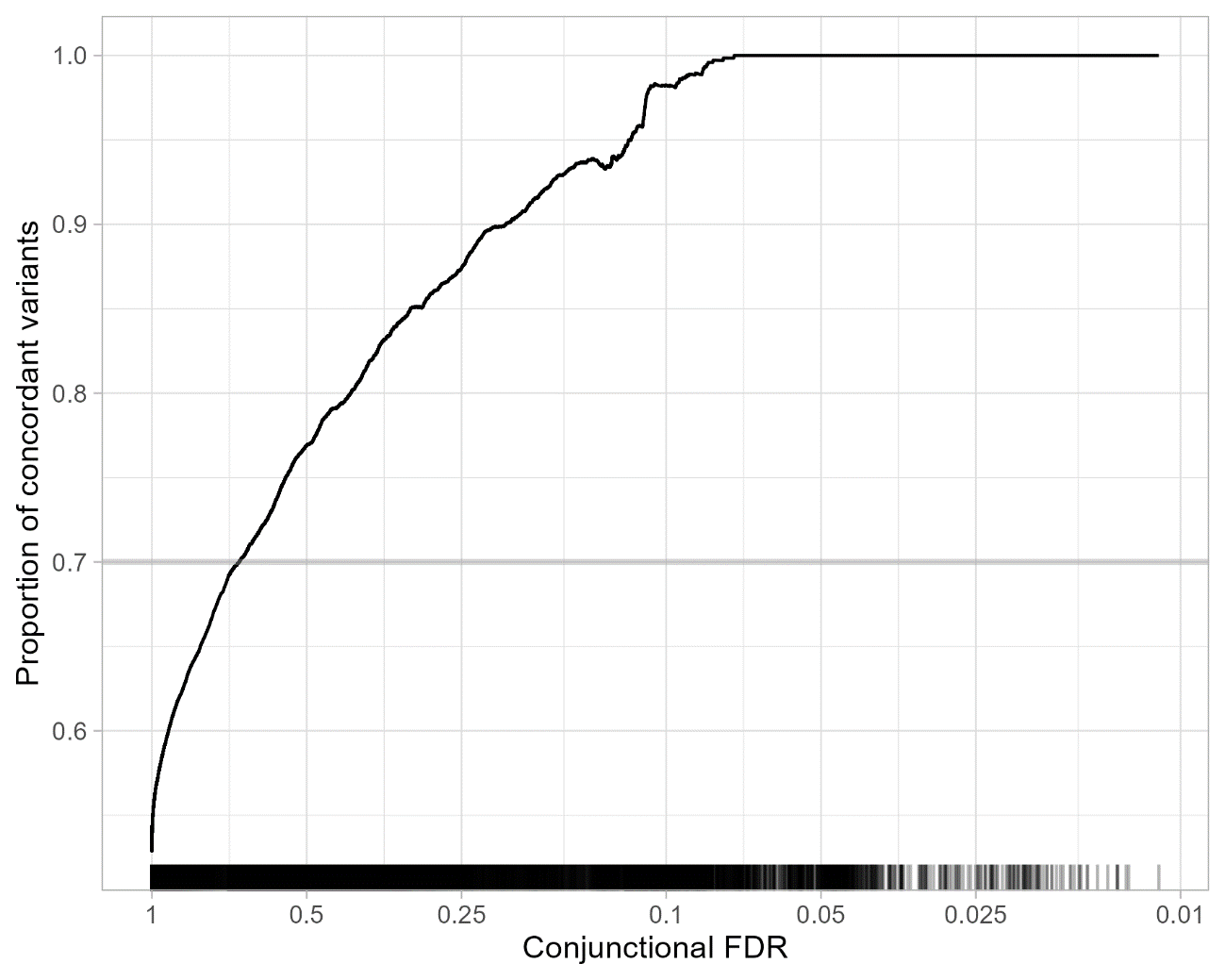


**Supplementary figure 6. Spatiotemporal gene expression pattern of 6 genes nearest to the shared loci between SCZ and AN across 16 brain regions and 11 developmental time points**

Mean expression difference between CELSR1 (A), NEGR1 (B), RELN (C), NCAM1 (D) , SOX5 (E) and TSNARE1 (F) versus 52,376 background genes with non-zero expression in BrainSpan dataset is illustrated in color ranging from red (high expression) to blue (low expression), respectively. The gene expression values are log2-transformed, and the differences in transformed expression values are centered and scaled across 16 brain regions at each time point. The color represents the relative differential expression in a brain region among all 16 brain regions at a specific time point. Brain regions are clustered using unsupervised hierarchical cluster analysis. VFC = ventrolateral prefrontal cortex ; V1C = primary visual cortex; OFC =The orbitofrontal cortex; A1C = primary auditory cortex; IPC = inferior parietal cortex; S1C = primary somatosensory cortex ; M1C = primary motor cortex; ITC = inferolateral temporal cortex; STC = superior temporal cortex; MD = mediodorsal nucleus of thalamus; MFC = medial prefrontal cortex; ACC = anterior cingulate cortex; DFC = dorsolateral prefrontal cortex; STR = striatum; CBC = cerebellum; HIP = hippocampus; AMY = amygdala.


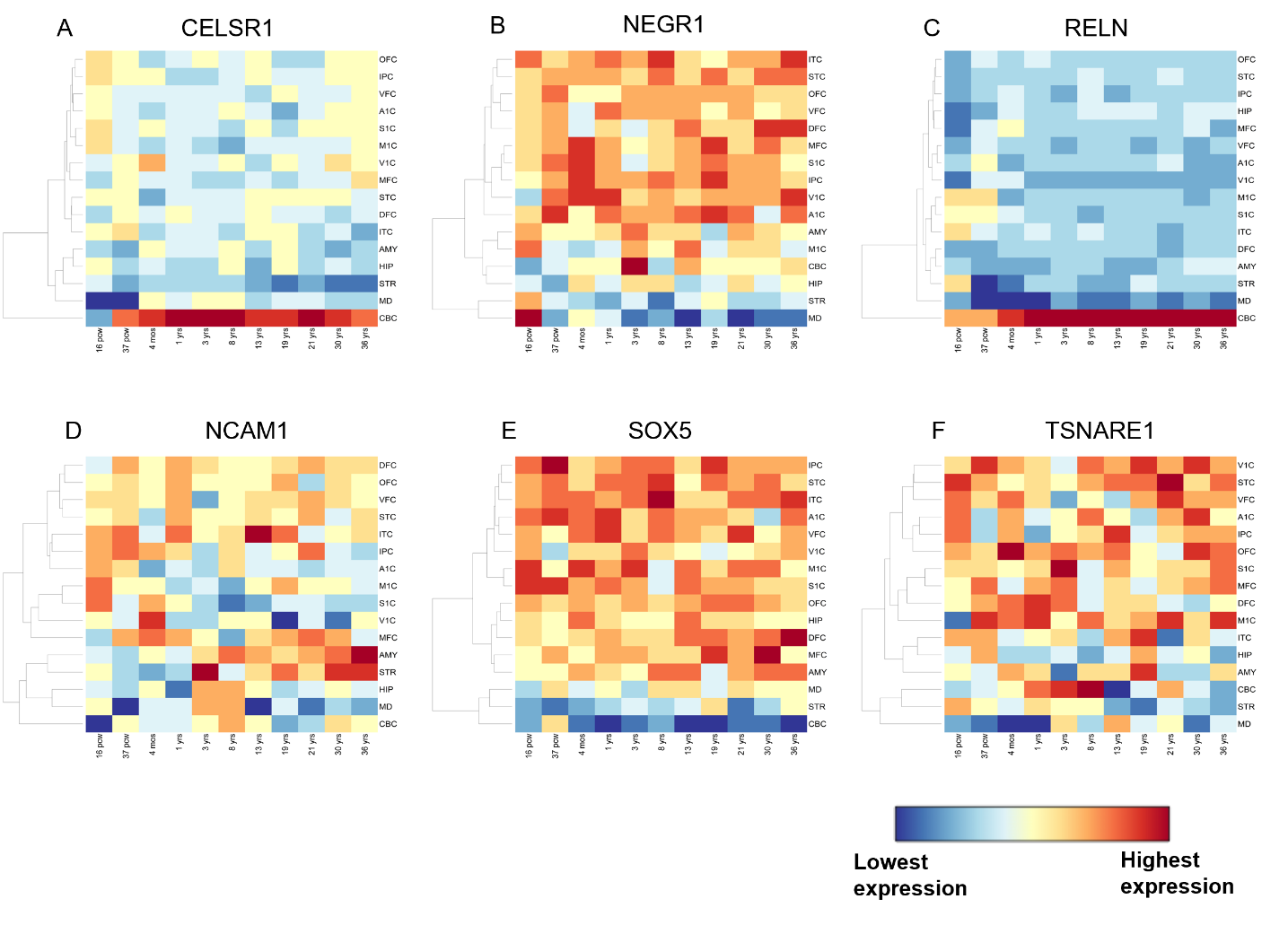

Supplement: sbae087_suppl_Supplementary_Material [file sbae087_suppl_supplementary_material.zip › Supplement_text _20240327.docx]
